# Understanding the S_N_2 Versus E2 Competition

**DOI:** 10.1002/chem.202501810

**Published:** 2025-06-24

**Authors:** Pascal Vermeeren, Thomas Hansen, Trevor A. Hamlin, F. Matthias Bickelhaupt

**Affiliations:** ^1^ Department of Chemistry and Pharmaceutical Sciences, AIMMS Vrije Universiteit Amsterdam De Boelelaan 1108 Amsterdam 1081 HZ The Netherlands; ^2^ Institute of Molecules and Materials Radboud University Heyendaalseweg 135 Nijmegen 6525 AJ The Netherlands; ^3^ Department of Chemical Sciences University of Johannesburg Auckland Park Johannesburg 2006 South Africa

**Keywords:** elimination reactions, Lewis base, reactivity, solvation, substitution reactions

## Abstract

Bimolecular nucleophilic substitution (S_N_2) and base‐induced elimination (E2) generally compete, and it is, therefore, essential to be in control of this competition in synthetic organic chemistry. Herein, we establish guiding principles based on quantitative molecular orbital (MO) theory and the activation strain model to understand and tune the competition between the S_N_2 and E2 reactions. We discuss the role of key factors, such as the nature of the Lewis base, leaving group, substrate structure, and the solvent. To this end, we introduce the concepts of *characteristic distortivity*, *transition state acidity*, *intrinsic nucleophilicity*, and *apparent nucleophilicity*. These intuitive concepts equip chemists with conceptual tools to better understand and design reactions for organic synthesis.

## Graphical abstract

We provide an intuitive framework rooted in quantitative molecular orbital theory to understand the factors that govern the competition between the S_N_2 and E2 reaction pathways. The guiding concepts are: *characteristic distortivity*, *transition state acidity*, *intrinsic nucleophilicity*, and *apparent nucleophilicity*.

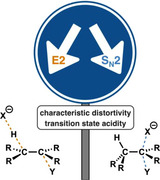

## 1 Introduction

**Scheme 1:**
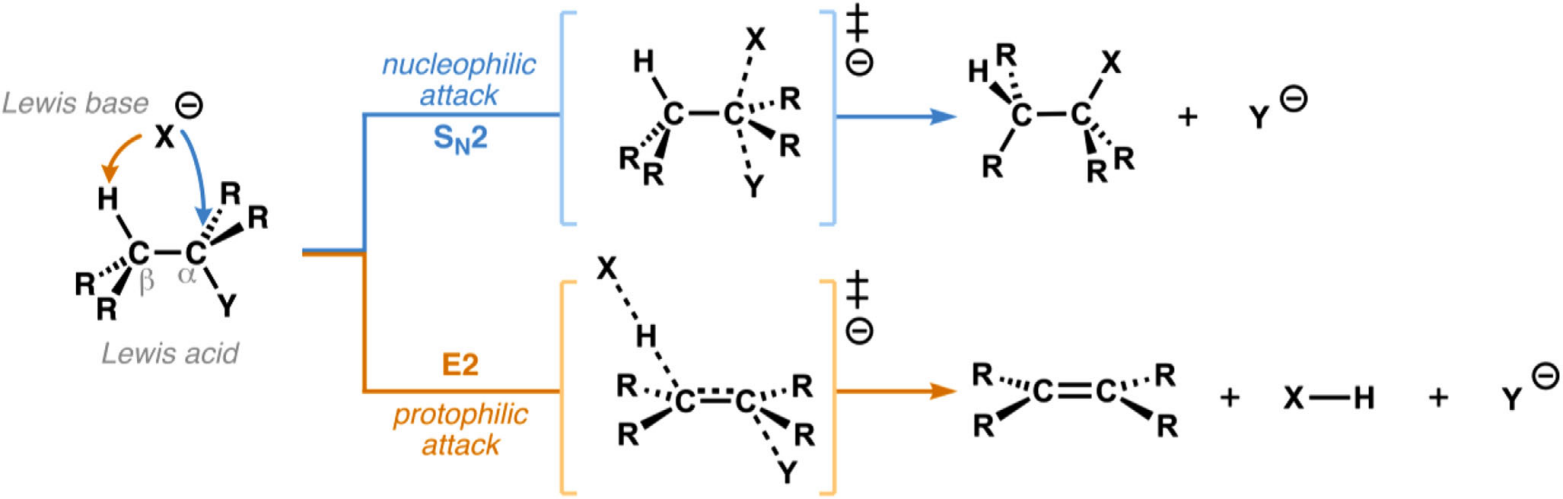
Generic S_N_2 and E2 pathways.

The bimolecular nucleophilic substitution (S_N_2) and base‐induced elimination (E2) reactions are fundamental pillars of organic chemistry, playing an essential role in the synthesis of many pharmaceuticals, flavors, fragrances, materials, and fine chemicals.^[^ ^1^ ^]^ In S_N_2 reactions, a Lewis base acts as a nucleophile, attacking the α‐carbon center and displacing the leaving group. In contrast, E2 reactions involve a Lewis base, acting as a protophile (Brønsted base), abstracting a proton from the β‐carbon center while simultaneously inducing the departure of a leaving group from the α‐position. In principle, the two mechanisms inherently compete whenever β‐hydrogens are present in the substrate bearing the leaving group (Lewis acid). Therefore, careful system tuning is required to direct the reaction toward the desired pathway and minimize undesired side reactions (Scheme 1). The pivotal role of these reactions has sparked numerous experimental^[^ ^2^ ^]^ and theoretical^[^ ^3^ ^]^ studies into how these processes proceed and compete in the gas phase and in solution, yielding valuable insights into the nature of these reaction mechanisms and their interplay. For example, Bierbaum found experimentally that in the gas phase, HO^−^ reacts via an E2 pathway with a set of alkyl halides, while HS^−^ follows an S_N_2 mechanism.^[^ ^2n^ ^]^ In general, strong Lewis bases act as protophiles and favor the E2 pathway, while weaker Lewis bases behave as nucleophiles and promote S_N_2 reactions.

## 2 Guiding Concepts for Understanding the S_N_2 Versus E2 Competition

The competition between S_N_2 and E2 reactions can be understood and rationalized using two new key concepts we developed within the framework of quantitative molecular orbital (MO) theory and the activation strain model^[^ ^4^ ^]^: *characteristic distortivity* and *transition state acidity*.^[^ ^5^ ^]^ Both these factors play a pivotal role in determining whether a reaction will follow the S_N_2 or E2 pathway. Importantly, these concepts are not just mechanistic distinctions but help to rationalize and explain how electronic and structural factors influence the behavior of Lewis bases and substrates in different scenarios. Understanding these concepts provides insight into the S_N_2 and E2 reaction mechanisms and allows for better prediction of outcomes when designing chemical processes involving these reaction pathways.

**Figure 1:**
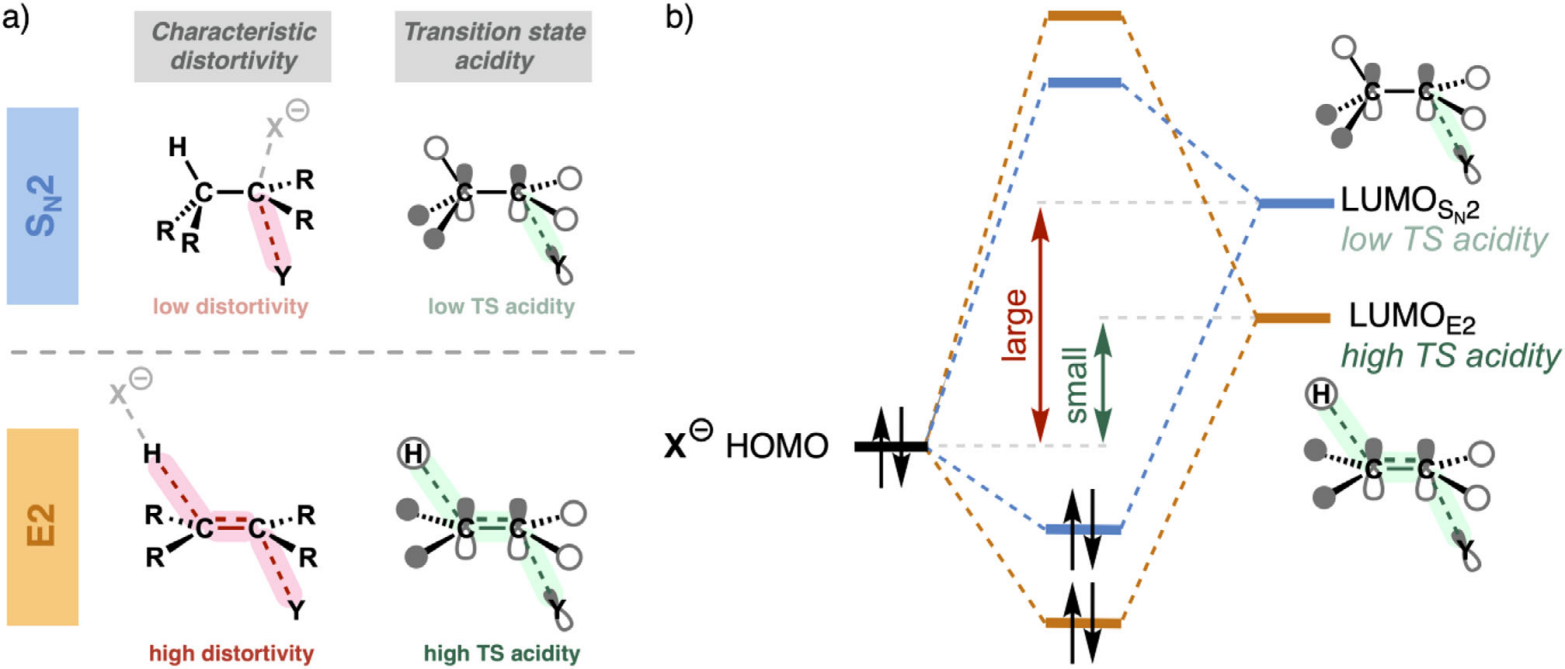
a) The concepts of *characteristic distortivity* and *transition state acidity*, applied to understanding the S_N_2 versus E2 competition. b) Schematic molecular orbital diagram displaying the effect of the transition state acidity on the Lewis acid–base interaction between Lewis base and substrate.

*Characteristic distortivity* refers to the degree of structural reorganization that occurs throughout the reaction from the reactants to the transition state (Figure 1a). In S_N_2 reactions, where only the bond between the α‐carbon and the leaving group (C^α^─Y) is broken, the distortivity is relatively low, resulting in less structural deformation in the transition state. In contrast, E2 reactions are characterized by breaking both the C^α^─Y bond and the stronger C^β^─H bond. This leads to a significant structural distortion and thus a more destabilizing activation strain in the transition state. In other words, the S_N_2 reaction is inherently associated with a lower characteristic distortivity, which is energetically favorable due to less activation strain, whereas the E2 reaction is inherently associated with a higher characteristic distortivity, which is energetically unfavorable due to more activation strain. Strong Lewis bases, such as alkoxides and hydroxide,^[^ ^6^, ^7^ ^]^ are typically reactive enough to overcome the higher distortivity of the E2 pathway and, therefore, often prefer to react as protophiles (vide infra).

**Figure 2:**
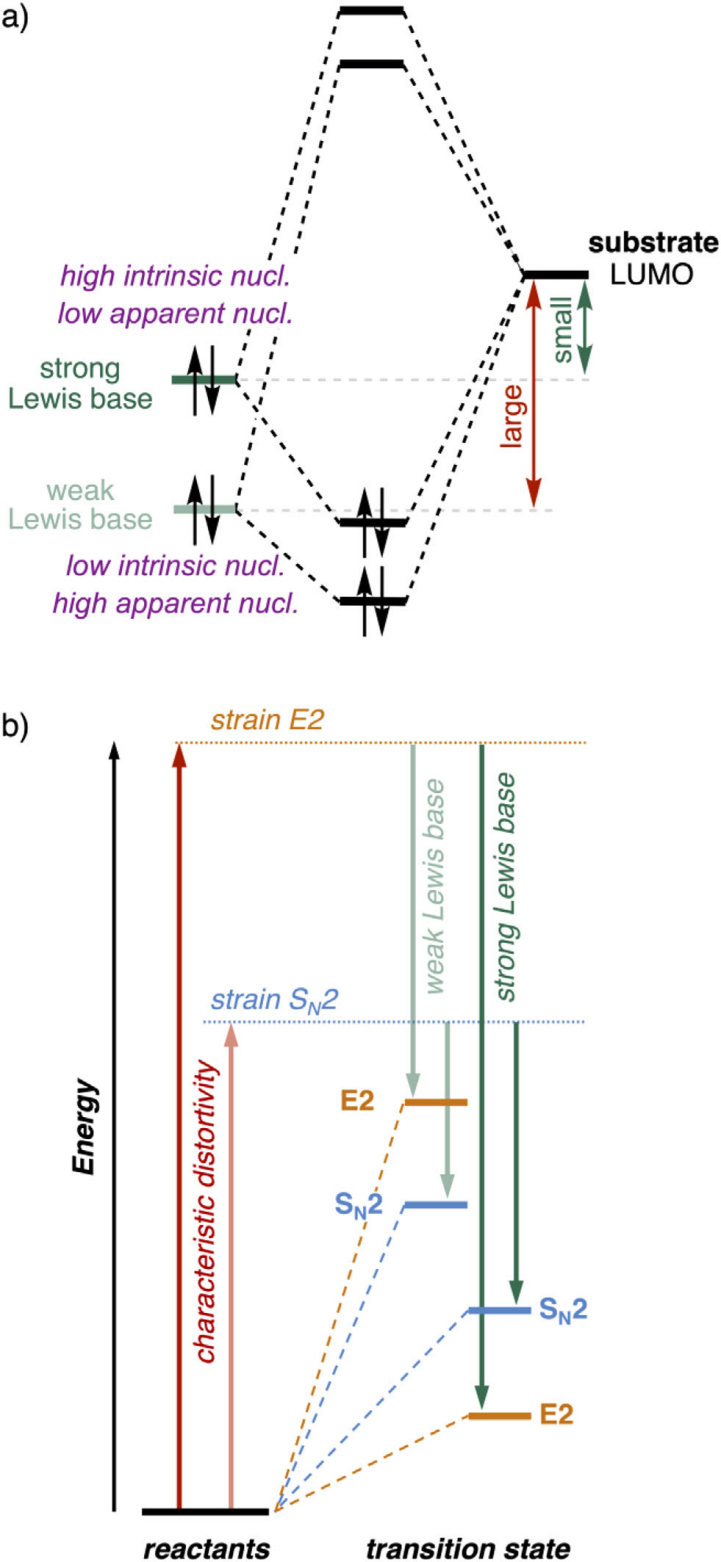
a) Effect of the basicity on the *intrinsic nucleophilicity* of the Lewis base engaging in a Lewis acid–base interaction with the substrate. Nucl. = nucleophilicity. b) Schematic activation strain diagram for the four transition states of the S_N_2 and E2 reactions of a weak and strong Lewis base (i.e., low‐ and high‐energy HOMO) with the substrate.

The concept of *transition state acidity* is also crucial in explaining why strong Lewis bases tend to favor the E2 mechanism. In the S_N_2 transition state, the substrate's lowest unoccupied molecular orbital (LUMO) loses antibonding character across the α‐carbon–leaving group (C^α^─Y) bond as this bond stretches. Consequently, the LUMO drops in energy, effectively enhancing the substrate's Lewis acidity. This electronic effect stabilizes the Lewis acid–base interaction with the highest occupied molecular orbital (HOMO) of the nucleophile. However, in the E2 transition state, both the C^β^─H and C^α^─Y bonds are broken, which leads to an even larger reduction of antibonding character in the LUMO of the Lewis acidic substrate, making it even more Lewis acidic. This amplified Lewis acidity allows strong Lewis bases to engage in a more stabilizing Lewis acid–base interaction that can overcome the unfavorably high characteristic strain for elimination, favoring the E2 over the S_N_2 pathway (Figures 1b, 2). Only weaker Lewis bases, such as thiolates and acetate,^[^ ^6^, ^7^ ^]^ will react as a nucleophile because in the regime of weak interactions, the activation strain remains as the dominant factor, and it favors the less distortive S_N_2 pathway (vide infra).

## 3 Effect of the Lewis Base and Leaving Group

Next, we use our guiding concepts to explain how changing the Lewis base can steer its behavior to act as a nucleophile or as a protophile, to induce an S_N_2 or an E2 reaction, respectively. In principle, both pathways benefit from a stronger Lewis base, that is, a base with a higher‐energy HOMO, because this leads to a more stabilizing Lewis acid–base interaction with the substrate and, therefore, a lower reaction barrier (Figure 2a). To facilitate the understanding of the S_N_2 versus E2 competition, we have introduced the new concepts of *intrinsic nucleophilicity* and *apparent nucleophilicity*.^[^ ^5^ ^]^

The basicity of the Lewis base, that is Lewis base strength, determines its *intrinsic nucleophilicity* (or intrinsic protophilicity) and hence its ability to engage in both S_N_2 and E2 reactions. When going from a strong to a weak Lewis base, its basicity, that is proton affinity, decreases. This has implications for its ability to react with the substrate, as it becomes both a worse nucleophile and worse protophile, in the sense that it ultimately leads to both higher barriers for S_N_2 (nucleophilic attack) and for E2 (protophilic attack) reactions. This reduced intrinsic nucleophilicity originates from the fact that the highest unoccupied molecular orbital (HOMO) of the Lewis base, which engages in a stabilizing interaction with the lowest unoccupied molecular orbital (LUMO) of the substrate, becomes more stable when going from a strong to a weak Lewis base (Figure 2a).^[^ ^8^ ^]^ The lowering of the Lewis base's HOMO results in a larger HOMO–LUMO orbital energy gap and hence weaker Lewis acid–base interaction between the Lewis base and the substrate. This, ultimately, gives rise to the increase of the reaction barrier.

Although the reaction barrier of both the S_N_2 and E2 pathways increases when moving to a weak Lewis base, there is a switch in preferred reaction pathway (Figure 2b). This is because the former pathway needs a more stabilizing interaction to overcome the unfavorably high characteristic strain associated with breaking two bonds (E2) instead of only one (S_N_2). Thus, while a strong Lewis base prefers the E2 reaction pathway, a weaker Lewis base favors the S_N_2 reaction pathway. This is what we call the *apparent nucleophilicity* of a Lewis base, which explains why weaker bases, despite their lower intrinsic nucleophilicity, tend to favor the S_N_2 over the E2 pathway. As mentioned above, weaker bases cannot overcome the high characteristic activation strain associated with the significant characteristic distortivity required for E2 reactions. As mentioned, during the E2 pathway, two bonds (C^β^─H and C^α^─Y) must be broken. It requires a strong Lewis base to overcome this higher distortivity by engaging in a strong Lewis acid–base interaction with the substrate. In contrast, the S_N_2 pathway involves breaking only the C^α^─Y bond, requiring less structural deformation and making it accessible to weaker Lewis bases. Thus, even with reduced intrinsic nucleophilicity, weaker Lewis bases more readily follow the S_N_2 pathway due to the less characteristic distortivity of the transition state along this reaction channel.

To summarize, the Lewis acid–base interaction between the Lewis base and the substrate in the transition state determines the outcome of the S_N_2 versus E2 competition: (i) in the case of a weak Lewis acid–base interaction, that is, when the Lewis base is weak (low basicity), the characteristic distortivity determines the barrier and this factor is always in favor, that is less destabilizing, for the S_N_2 pathway; (ii) in the case of a strong Lewis acid–base interaction, that is, when the Lewis base is strong (high basicity), the strong Lewis acid–base interaction overrules the corresponding characteristic distortivity and determines the preferred reaction pathway. This factor is always in favor, that is more stabilizing, for the structurally more deformed E2 pathway along which the substrate acquires a higher Lewis acidity. These findings show that the nucleophilic or protophilic behavior of a Lewis base toward a Lewis‐acidic substrate is fundamentally codetermined by the latter.

A special class of Lewis bases are α‐nucleophiles, featuring a lone pair‐bearing heteroatom adjacent to the nucleophilic center, which, in certain cases, engage in a dramatically enhanced S_N_2 and E2 reactivity compared to their parent normal nucleophile analog.^[^ ^9^ ^]^ This phenomenon of enhanced reactivity is called the α‐effect and originates from the fact that these α‐nucleophiles experience less steric repulsion with the substrate.^[^ ^10^ ^]^ The adjacent heteroatom of α‐nucleophiles can have two effects: First, the adjacent heteroatom can polarize orbital density away from the nucleophilic center resulting in a smaller spatial HOMO lobe on the nucleophilic center, and less steric (Pauli) repulsion with the substrate. Second, despite its significantly smaller lobe on the nucleophilic center, the α‐nucleophile HOMO can still engage in a strongly stabilizing Lewis acid–base interactions, similar to its parent normal nucleophile, because this HOMO is antibonding between the two hetero atoms and therefore has a high orbital energy. This situation results in a smaller HOMO–LUMO orbital energy gap and hence a more stabilizing orbital interaction.

Besides the Lewis base, the leaving group of the substrate also plays a role in the S_N_2 and E2 pathways. A weaker carbon–leaving group bond, that is a lower carbon–leaving group bond enthalpy,^[^ ^11^ ^]^ corresponds to a better leaving group.^[^ ^5^ ^]^ The weakening of the carbon–leaving group bond manifests itself in a reduced activation strain along both the S_N_2 and E2 reaction pathways, while the interaction between the Lewis base and the substrate remains essentially constant. Changing the leaving group, therefore, has little effect on the intrinsic S_N_2 versus E2 competition.^[^ ^5a^ ^]^

## 4 Effect of Solvation

**Figure 3:**
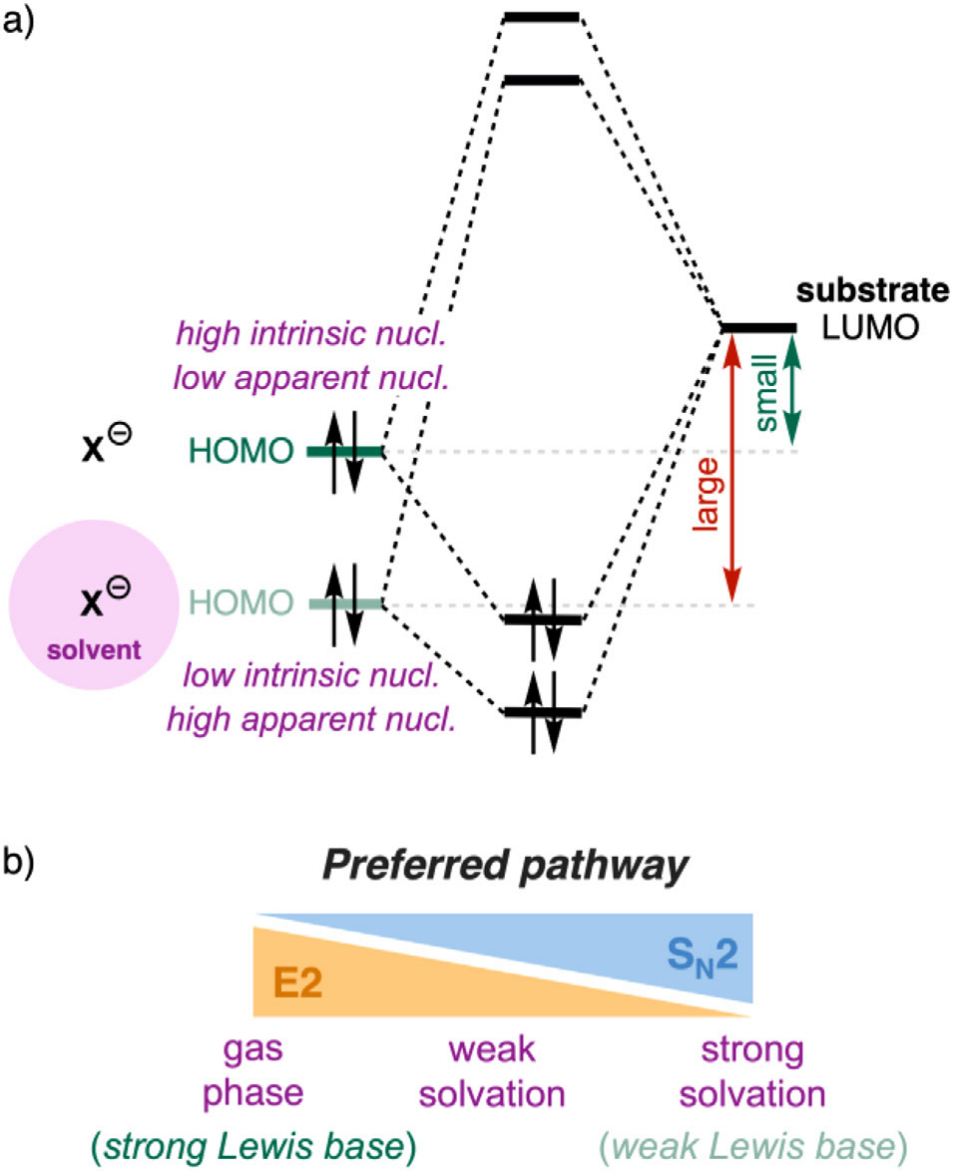
a) Influence of solvation on *intrinsic* and *apparent nucleophilicity* of the Lewis base basicity and Lewis acid–base interaction with the substrate. Nucl. = nucleophilicity. b) S_N_2 versus E2 competition going from the gas phase to weak solvation to strong solvation.

How does solvation affect the competition between the S_N_2 and E2 pathways?^[^ ^12^ ^]^ As a Lewis base moves from the gas phase into solution, its basicity, that is Lewis base strength, decreases due to interactions with the solvent. This effect raises both the S_N_2 and E2 reaction barrier for (anionic) Lewis bases.^[^ ^13^ ^]^ The solvent effectively acts as a weak Lewis acid that stabilizes the electron density of the solute, that is, it stabilizes the occupied orbitals. In particular, it stabilizes the Lewis base's HOMO, reducing its intrinsic nucleophilicity (Figure 3a). As a result, the solvated Lewis base is a weaker base and interacts in a less stabilizing manner with the substrate, giving rise to a higher reaction barrier compared to the analogous reaction in the gas phase.^[^ ^14^ ^]^

Solvation not only raises the barriers but also influences the competition between the S_N_2 and E2 reactions, which can again be rationalized using the guiding concepts proposed earlier. In the gas phase, strong Lewis bases can strongly interact with the substrate and overcome the higher deformation energy of the E2 pathway. In solution, however, the same Lewis base is weaker, and this, shifts the preference toward the S_N_2 pathway (Figure 2b). The solvated Lewis base cannot engage in a sufficiently strong Lewis acid–base interaction with the substrate and hence cannot overcome the high characteristic strain associated with the E2 pathway. For example, Jones and Ellison showed that, in the gas phase, methoxide and 1‐bromopropane react exclusively via E2, in stark contrast with the reactivity of alcoholic sodium ethoxide toward the same substrate which proceeds 91% via S_N_2.^[^ ^15^, ^16^ ^]^ In a more recent example, Bierbaum and Westaway showed experimentally that gas‐phase reactions of cyanide with alkyl iodides proceed predominantly via S_N_2, but with a minor contribution from E2, in contrast to solution‐phase conditions where both protic and aprotic solvents facilitate exclusively S_N_2 reactivity.^[^ ^2h^ ^]^ The magnitude of this shift from E2 to S_N_2 depends on the nature and polarity of the solvent. In polar (protic) solvents, such as water or DMSO, the shift is more pronounced than in less polar solvents, such as alcohols, or apolar solvents, such as toluene or DCM.^[^ ^1^, ^15^ ^]^

## 5 Effect of the Substrate Structure

Another important factor in the competition between the S_N_2 and E2 pathways is the structure of the substrate. Cyclic substrates featuring the leaving group in their ring can shift the preference from one pathway to the other in the S_N_2 versus E2 competition, depending on their ring size.^[^ ^17^ ^]^ Reducing the ring size accelerates the S_N_2 reaction, making it significantly faster than for the acyclic analogs. Conversely, the E2 reaction, especially with strong Lewis bases, slows down upon decreasing the ring size. These opposing reactivity trends cause a shift in the favored pathway when using strong Lewis bases: for large cyclic substrates, the E2 pathway dominates, whereas, for smaller cyclic substrates, the S_N_2 pathway becomes the preferred pathway. Weak Lewis bases always favor the less distortive S_N_2 reaction.

**Figure 4:**
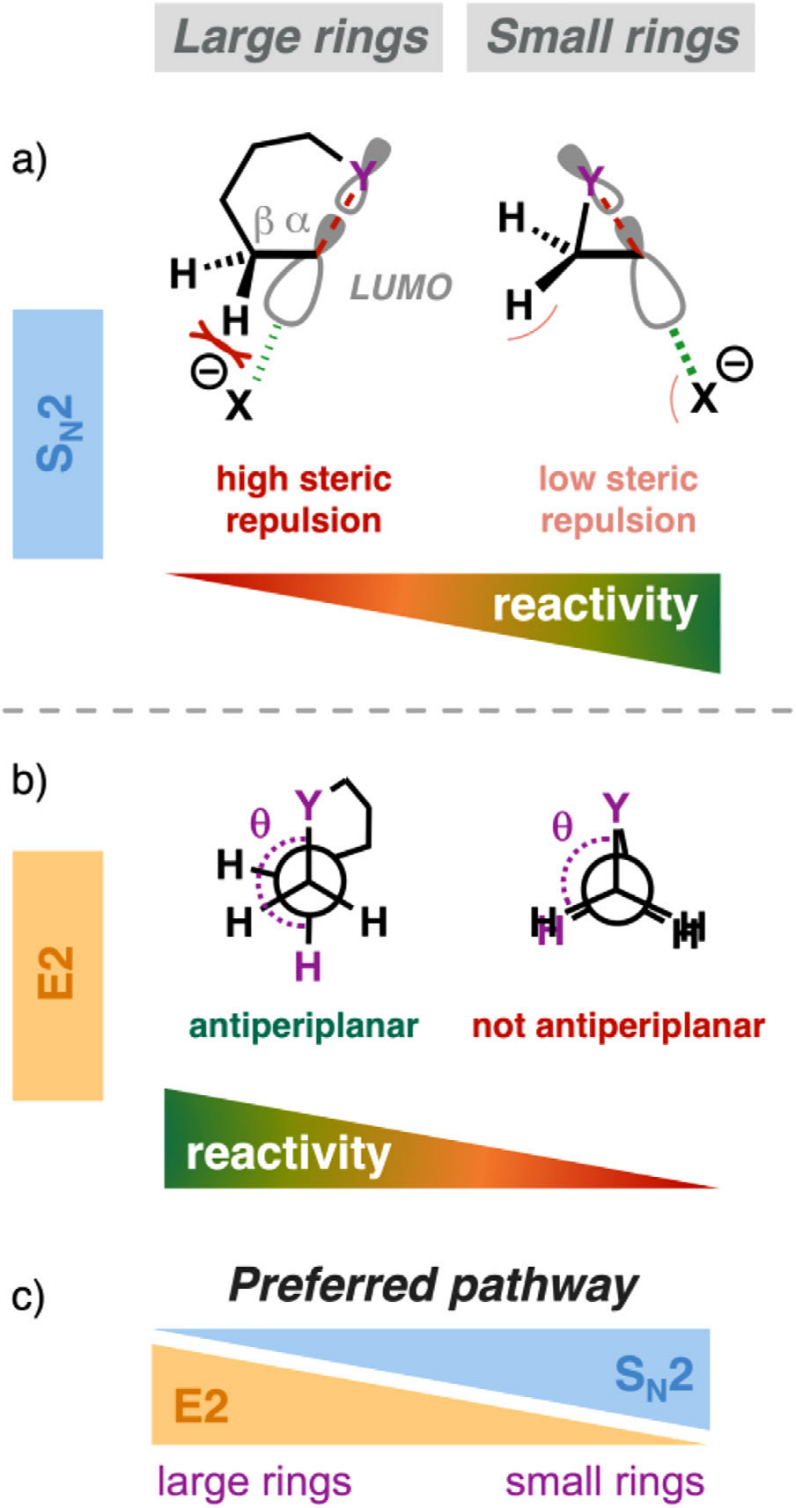
The effect of ring size of cyclic substrates on the reaction barrier along the a) S_N_2 and b) E2 pathway, and c) the S_N_2 versus E2 competition.

Reducing the substrate's ring size accelerates the S_N_2 pathway by strengthening the Lewis acid–base interaction with the nucleophile due to less steric repulsion.^[^ ^18^ ^]^ In large‐ring substrates and acyclic analogs, the approaching nucleophile experiences significant steric repulsion with the C^α^─C^β^ and C^β^─H bonds as it interacts with the substrate's LUMO (Figure 4a). In contrast, small rings pull the C^α^─Y and C^α^─C^β^ bonds closer together due to their shorter tethered chains, leaving the LUMO of the substrate less shielded by the C^α^─C^β^ and C^β^─H bonds. This lowers steric repulsion between the incoming nucleophile and the substrate, strengthening the mutual interaction. For this reason, the S_N_2 barrier becomes lower in the case of smaller rings. This trend is further amplified by the favorable release of ring strain for the small rings, which shows up in a less destabilizing activation strain compared to larger analogs.

In contrast, for the E2 pathway, the Lewis acid–base interaction is weakened for smaller rings (Figure 4b), because the inherent structural constraints hamper the substrate from adopting an optimal antiperiplanar arrangement between the C^β^─H and the C^α^─Y bonds (Y─C^α^─C^β^─H θ = 180°). As a result, the σ∗_C─H_ and σ*_C─Y_ features of the LUMO are less coupled, which slow down the lowering of this acceptor orbital as the proton is abstracted. Consequently, the substrate is effectively less acidic, and there is a weaker Lewis acid─base interaction with the Lewis base.

These contrasting reactivity trends impact the S_N_2 versus E2 competition. Weak Lewis bases favor the S_N_2 pathway, independent of the size of the substrate's ring. In contrast, strong Lewis bases exhibit a switch in preference from E2 for acyclic and cyclic substrates with a larger ring to S_N_2 for substrates with a small ring. For the larger ring substrates, strong Lewis bases can effectively overcome the high characteristic distortivity associated with the E2 mechanism, due to the strong Lewis acid–base interaction with the substrate. However, for smaller rings, the Lewis base cannot engage in a sufficiently stabilizing Lewis acid–base interaction with the substrate because its LUMO remains at a higher, less favorable orbital energy. Hence, the interaction is unable to overcome the higher characteristic strain of the E2 pathway, shifting the system's reactivity toward the less distortive S_N_2 pathway.

## 6 S_N_2 Versus S_N_2' Competition

When substrates contain an allylic group adjacent to the C─Y leaving group, there is a scenario where S_N_2 competes with the allylic S_N_2' pathway.^[^ ^19^ ^]^ Similar to the situation for the E2 pathway in the S_N_2 versus E2 competition, the allylic S_N_2' pathway – characterized by the allylic rearrangement in addition to breaking of the C─Y bond — exhibits a higher characteristic distortivity than the S_N_2 pathway.^[^ ^20^ ^]^ The higher distortivity of the S_N_2' pathway stabilizes the substrates' LUMO, resulting in a substrate with an effectively higher transition state acidity. For strong Lewis bases, this leads to a sufficiently stabilizing Lewis acid–base interaction that can overcome the higher characteristic strain of the S_N_2' pathway. Conversely, for weaker Lewis bases, the Lewis acid–base interaction is not strong enough to overcome the higher activation strain of the allylic S_N_2' pathway, and, thus, the less distortive S_N_2 pathway becomes the dominant pathway.

## 7 Summary and Outlook

This concept article presents a framework rooted in quantitative molecular orbital (MO) theory and the activation strain model to elucidate how variations in the Lewis base, leaving group, substrate structure, and solvent can be used to tune the rate of S_N_2 and E2 pathways and their mutual competition. We have introduced four key guiding concepts that play a pivotal role in determining whether a reaction will follow the S_N_2 or E2 pathway:

▪ ***Characteristic distortivity***: The degree of structural deformation that occurs along a specific reaction pathway. A higher characteristic distortivity translates into a higher, more destabilizing characteristic strain.
▪ ***Transition state acidity***: The Lewis acidity of the substrate along the specific reaction pathways, that is stability of the substrate's LUMO. A higher transition state acidity leads to a more stabilizing interaction with the attacking Lewis base.
▪ ***Intrinsic nucleophilicity***: The ability of a Lewis base to achieve a high or low barrier for the S_N_2 pathway. The intrinsic nucelophilicity is higher if the Lewis base's HOMO is higher in energy, that is if it is more basic.
▪ ***Apparent nucleophilicity***: The extent to which a Lewis base prefers to act as a nucleophile and favor the S_N_2 rather than act as a protophile and follow the E2 pathway. This property depends on both the Lewis base and the substrate. In general, the apparent nucleophilicity *increases* as the basicity *decreases*.

In the competition between S_N_2 and E2 pathways, the E2 reaction inherently has a higher characteristic distortion (two bonds vs. one bond breaking) and thus more destabilizing characteristic strain than the S_N_2 reaction. This structural deformation leads to a higher transition state acidity (more acidic substrate) for the E2 pathway compared to the S_N_2 reaction. Strong Lewis bases can overcome the associated high strain of the E2 by effectively interacting with the more acidic substrate. In contrast, weak Lewis bases favor the less distorted S_N_2 pathway, as their weaker interaction is insufficient to surmount the higher activation strain of the E2 pathway. This explains why solvation shifts the reaction preference toward S_N_2: solvation stabilizes the base and diminishes its basicity.

The guiding concepts proposed herein can also be applied to other properties that affect the S_N_2 and E2 competition, such as the bulkiness of the Lewis base or the pH of the medium, ^[^ ^21^ ^]^ as well as to other competing mechanisms. For example, in the case of oxidative addition of carbon–halogen bonds to palladium, either via S_N_2 attack of the metal at the carbon atom (Walden inversion) or via direct oxidative insertion (retention of configuration).^[^ ^22^ ^]^ Future studies will further explore our concepts in yet other reaction mechanisms.

## Conflict of Interest

The authors declare no conflict of interest.

## Data Availability

Data sharing is not applicable to this article as no new data were created or analyzed in this study.
